# HIV-1 Drug Resistance Genotyping in Resource Limited Settings: Current and Future Perspectives in Sequencing Technologies

**DOI:** 10.3390/v13061125

**Published:** 2021-06-11

**Authors:** Sontaga Manyana, Lilishia Gounder, Melendhran Pillay, Justen Manasa, Kogieleum Naidoo, Benjamin Chimukangara

**Affiliations:** 1 National Health Laboratory Service, Department of Virology, School of Laboratory Medicine and Medical Sciences, University of KwaZulu-Natal, Durban 4058, South Africa; Gounder@ukzn.ac.za (L.G.); melendhra.pillay@nhls.ac.za (M.P.); ChimukangaraB@ukzn.ac.za (B.C.); 2 Department of Laboratory Medicine and Investigative Sciences, Faculty of Medicine and Health Sciences, University of Zimbabwe, Harare, Zimbabwe; jmanasa@uz.edu.zw; 3 Centre for the AIDS Programme of Research in South Africa (CAPRISA), Durban 4013, South Africa; Kogie.Naidoo@caprisa.org; 4 South African Medical Research Council (SAMRC), CAPRISA HIV-TB Pathogenesis and Treatment Research Unit, Durban 4013, South Africa

**Keywords:** HIV-1 drug resistance, Sanger sequencing, next generation sequencing, resource limited settings

## Abstract

Affordable, sensitive, and scalable technologies are needed for monitoring antiretroviral treatment (ART) success with the goal of eradicating HIV-1 infection. This review discusses use of Sanger sequencing and next generation sequencing (NGS) methods for HIV-1 drug resistance (HIVDR) genotyping, focusing on their use in resource limited settings (RLS). Sanger sequencing remains the gold-standard method for detecting HIVDR mutations of clinical relevance but is mainly limited by high sequencing costs and low-throughput. NGS is becoming a more common sequencing method, with the ability to detect low-abundance drug-resistant variants and reduce per sample costs through sample pooling and massive parallel sequencing. However, use of NGS in RLS is mainly limited by infrastructure costs. Given these shortcomings, our review discusses sequencing technologies for HIVDR genotyping, focusing on common in-house and commercial assays, challenges with Sanger sequencing in keeping up with changes in HIV-1 treatment programs, as well as challenges with NGS that limit its implementation in RLS and in clinical diagnostics. We further discuss knowledge gaps and offer recommendations on how to overcome existing barriers for implementing HIVDR genotyping in RLS, to make informed clinical decisions that improve quality of life for people living with HIV.

## 1. Introduction

In an effort to end the HIV epidemic by 2030, the UNAIDS 95-95-95 goals aim to ensure that 95% of all people living with HIV know their status, 95% of all people diagnosed receive sustained antiretroviral treatment (ART), and 95% of all people receiving ART have sustainable viral suppression [1,2]. HIV-1 drug resistance (HIVDR) remains a major threat to achieving these targets, particularly in achieving the third target of 95% viral suppression [3]. HIVDR genotyping before ART initiation and at ART failure improves effectiveness of subsequent treatment [4,5], but its use in resource limited settings (RLS) is often restricted by costs. Although Sanger sequencing remains the preferred clinical diagnostic method for HIVDR genotyping, next generation sequencing (NGS) methods are becoming more affordable but their use in the therapeutic management of patients on ART is yet to be established [6].

Sanger sequencing is a population-based method that uses a di-deoxy chain termination chemistry to generate a single consensus sequence representative of the most common bases at each nucleotide position [7]. This, however, means that Sanger sequencing does not reliably detect mutations that are less represented within the viral pool, also known as low-abundance drug-resistant variants (LA-DRVs) [8,9]. Despite that, HIVDR mutations detected by Sanger sequencing have been shown to predict treatment response, making it a reliable method for use in making clinical decisions [10]. On the other hand, NGS is more sensitive, having the ability to detect LA-DRVs (i.e., viral variants at <20%) [11], and enabling quantitative detection of HIVDR mutations [12]. However, there remains a dearth of knowledge around the clinical significance of LA-DRVs on ART effectiveness [13], although a high concordance with Sanger sequencing has been shown in detection of mutations at ≥20% frequency [6].

NGS also has the ability for massive parallel sequencing of individual input templates, generating incredible amounts of data per sequencing run [11,14]. Given its high-throughput and cost-efficiency through parallel sequencing and sample pooling, NGS is becoming a more common method for HIVDR genotyping [14,15]. In this review, we discuss the available and emerging Sanger sequencing and NGS methods for HIVDR genotyping, highlighting challenges for their use in RLS and for clinical diagnostics.

## 2. Overview of Sanger Sequencing and NGS Platforms

Sanger sequencing platforms have largely been dominated by Applied Biosystems, with instruments ranging from 3100- to 3700-series, as well as the SeqStudio (Applied Biosystems, Tustin, CA, USA) instrument [16]. The ABI 3730xl (Applied Biosystems, Tustin, CA, USA) genetic analyzer is the most robust of these instruments with high-throughput, scalability and flexibility. It can sequence fragments up to 900 bp in a single read and can process 96 reactions simultaneously and continuously for several sequencing plates at a time [17]. However, its throughput is nowhere comparable to NGS, which can produce millions of reads from a single sequencing run [18,19]. Moreover, NGS has several sequencing platforms from different manufacturers such as Illumina (Illumina, CA, USA), Thermo Fisher (Thermo Fisher Scientific, Waltham, MA, USA), Pacific Biosciences (PacBio) (PacBio, CA, USA) and Oxford Nanopore Technologies (ONT) (ONT, Oxford, UK). Illumina uses a sequencing-by-synthesis chemistry and is arguably one of the most widely used NGS platforms, offering flexibility in choice of instruments, with varying read-lengths and sequencing outputs [20]. Considering that HIVDR genotypic testing in RLS is mainly targeted on the HIV-1 *pol* gene, Illumina’s short read sequencing and lower instrument cost make it a front runner for HIVDR genotyping [21]. Table 1 provides a summary of the popular NGS platforms, highlighting their technical differences.

## 3. Sanger Sequencing and NGS for HIVDR Genotyping

Several in-house Sanger sequencing assays have been developed over the years for HIVDR genotyping. However, there is currently one commercially available Sanger sequencing assay approved by the US Food and Drug Administration (FDA) for HIVDR genotyping, namely ViroSeq HIV-1 Genotyping kit [23,24,25,26]. The kit is relatively expensive, costing approximately US$380 per test, making it less affordable for RLS [27]. This makes laboratories resort to developing low-cost in-house assays, and in some cases modifying commercial assays to reduce costs [28]. A personal view by Inzaule et al. previously estimated that the costs of HIVDR genotyping in-house assays range from US$47.50 to US$155, without labor costs [14], although these could vary further depending on costs of procuring laboratory reagents and consumables.

A collaborative partnership between the Southern African Treatment and Resistance Network (SATuRN) and not-for-profit manufacturers (Life Technologies/Centers for Disease Control and Prevention) previously developed a discounted HIVDR genotyping assay that reliably detects mutations in the HIV-1 protease (PR) and reverse transcriptase (RT) genes from plasma and dried blood spot samples by Sanger sequencing [14,29,30,31]. Such partnerships are essential to validate in-house HIVDR genotyping assays and make them commercially affordable for RLS. Table 2 shows a summary of published in-house HIVDR genotyping assays targeted for use in RLS.

**Figure 1:**
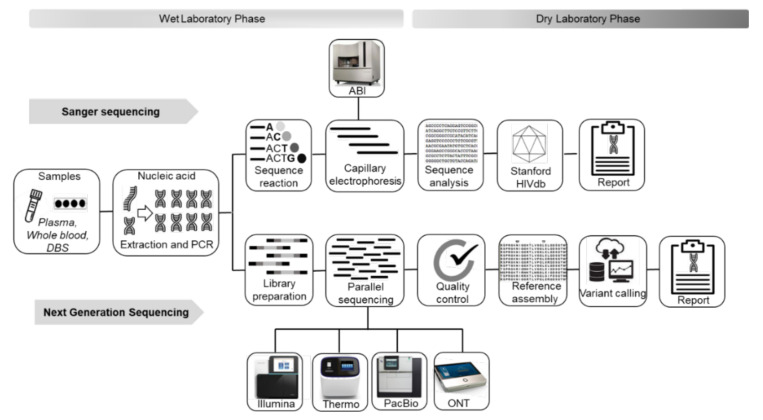
Summary comparison of Sanger sequencing and NGS HIVDR workflows.ABI, Applied Biosystems; DBS, dried blood spots; HIV db, HIV drug resistance database; ONT, Oxford Nanopore Technology; PacBio, Pacific Biosciences; PCR, polymerase chain reaction; Thermo, Thermo Fisher.

There is currently only one commercially available NGS assay approved by the FDA in November 2019 for clinical HIVDR genotyping, known as the Sentosa SQ HIV-1 Genotyping Assay [12,50]. This is a standardized, semi-automated and novel deep in vitro sequencing assay developed by Vela Diagnostics (Vela-Diagnostics, Humburg, Germany) for sequencing the combined HIV-1 PR, RT and integrase (IN) genes with minimal expertise [51]. The instrument alone costs ~US$400,000 with a per sample cost of around US$400, making it an expensive option for RLS. All other NGS methods for HIVDR genotyping are based on in-house assays developed to the convenience of the laboratories running the assays. However, most of these in-house assays are labor intensive and require additional quality control measures to lessen the effect of sequencing errors generated by NGS technologies. Figure 1 summarizes the Sanger and NGS workflows from sample to producing a report.

## 4. Advantages and Disadvantages of Sequencing Methods for HIVDR Genotyping

For over two decades Sanger sequencing has been shown to be a highly reproducible and interpretable method for diagnosing HIVDR in clinical settings [14,32,33]. This is because Sanger sequencing has been available long enough for use in HIVDR genotyping, producing high quality sequence data, with short turnaround times, and relatively simple workflows and data interpretation [14]. However, in addition to the inability of Sanger sequencing to reliably detect LA-DRVs [19,52], it has limited data throughput [53]. Also, there is less flexibility in choice of sequencing platforms, which is mainly limited to one manufacturer (i.e., Thermo Fisher). Moreover, Sanger sequencing requires standard molecular biology workflows and infrastructure which are mostly available in centralized laboratories in RLS [14]. This reduces its accessibility and affordability for HIVDR genotyping in RLS; hence, its preferred for use only after second-line ART failure [54].

In contrast, there is a wider range of NGS platforms supplied by various manufacturers, using different sequencing chemistries (Table 1) and achieving massive parallel sequencing [8]. This offers laboratories flexibility in choosing which assays to run but poses a challenge in reproducibility across various platforms. However, there is lack of validated NGS methods for clinical use, which is compounded by high sequencing errors and workflows that are labor intensive. NGS methods also require high infrastructure costs and high levels of expertise [14]. In addition, long-term investment in remote and/or cloud-based servers is required to keep up with the large amounts of data generated by NGS. The vast amounts of data also pose challenges in data analysis [55]. Individual laboratories often develop their own data analysis pipelines, making it difficult to normalize data quality across the board. Table 3 summarizes the strengths and weaknesses of Sanger sequencing and NGS in relation to their use in HIVDR genotyping [14].

## 5. Future Recommendations for HIVDR Genotyping and Knowledge Gaps

As new drugs to treat HIV-1 patients increase and extensive drug class resistance grows, HIVDR genotyping methods need to evolve to determine resistance to newer drug classes such as capsid inhibitors, entry inhibitors, nucleoside analogue reverse transcriptase translocation inhibitors and Rev inhibitors. For instance, as ART programs in RLS adopt use of integrase strand transfer inhibitors such as dolutegravir and possibly the long-acting injectable cabotegravir, sequencing of longer reads that cover all relevant HIV-1 viral genes (i.e., PR, RT, and IN) is required. Combined PCR amplification of these genes is difficult as they are spread apart. Genotyping PR and RT genes in a separate assay to the IN gene is an option; however, this doubles the HIVDR genotyping workload. Therefore, there is a need to design Sanger sequencing assays that detect all relevant mutations in a single amplicon, in order to simplify HIVDR genotyping for clinical diagnostics and research use in RLS.

Although there are several advantages of Sanger sequencing over NGS for clinical diagnostic use, further technical innovations are still required to make Sanger sequencing affordable and available in RLS. The high cost per sample remains a major drawback for implementation of HIVDR genotyping in RLS. NGS can reduce the per sample costs through sample pooling which, however, results in longer turnaround times. Centralizing NGS for HIVDR genotyping could help address the challenge of longer turnaround times and higher per sample costs. Subsidizing of instrument and consumable costs by manufacturers for RLS, together with governmental support will also go a long way in reducing costs and establishing NGS in RLS.

Requirements for expert bioinformatics analyses and high sequencing errors also remain major barriers for use of NGS in HIVDR genotyping. There is a need for international standards that guide NGS for HIVDR genotyping, as well as standardized data analysis pipelines to ensure reproducible, accurate and high-quality data outputs. This could be achieved through internal and external quality assurance programs [11], and validating in-house assays against those that are FDA approved, such as the Sentosa SQ HIV-1 Genotyping Assay. Moreover, validation of commonly used data analysis pipelines for HIVDR mutation calling, such as HyDRA, PASeq and MiCall, is required to address challenges with bioinformatics and data quality [12]. These data analysis pipelines commonly comprise filtering of low-quality data, detection and quantification of amino acid variants at known mutation positions.

HyDRA, PASeq and MiCall are freely available web-based data analysis pipelines that require only minimal bioinformatics expertise and infrastructure [55]. They all use the Stanford HIV drug resistance database (Stanford HIVdb) algorithm, allowing for easy HIV resistance interpretation [21]. HyDRA is compatible with both Illumina and Ion torrent sequencing data and it uses quality control and sequencing error models for data quality assurance [21]. Unlike HyDRA, PASeq is compatible with only Illumina sequencing data. It uses quality control, contamination control and APOBEC hypermutation detection for data quality assurance [21]. Similar to PASeq, MiCall is only compatible with Illumina sequencing data. It uses quality control and sequencing error models for data quality assurance [21]. Despite the minor differences in procedures for data processing and reporting, these analysis pipelines have shown comparable HIV resistance interpretation with less bias for variants at ≥5% threshold, although the clinical relevance of LADRVs (i.e., including those at ≥5%–<20% thresholds) require further investigations [21,55]. Therefore, these data analysis pipelines can drastically reduce quality control problems associated with NGS and have potential to produce consistent HIV resistance data that is easily and rapidly interpreted to inform clinical decisions.

In addition, understanding the clinical relevance of LA-DRVs detected by NGS remains elusive for use of NGS data in HIV-1 treatment clinical decisions [6]. The Sentosa HIV-1 Genotyping Assay detects up to 10% variant threshold, but the decision on which threshold accurately predicts treatment response is yet to be made, and requires international efforts [12]. As NGS platforms continue to improve with reduced sequencing error rates, the ability to sequence multiple samples in a single sequencing run and possibly automating data analysis will make NGS more feasible, accessible and affordable for HIVDR genotyping in RLS [21].

## 6. Conclusions

HIVDR remains the greatest barrier to sustainable viral suppression on ART, whilst cost remains the greatest barrier to HIVDR genotyping in RLS. Available in-house and commercial sequencing assays should aim to offer accessible and relevant cost-effective HIVDR genotyping that can be used to make clinical decisions. In this review, we emphasize the need for Sanger sequencing assays to adapt to dynamic HIV-1 treatment programs, to simplify and make HIVDR genotyping affordable. We also highlight that NGS has great potential to achieve low-cost HIVDR genotyping useful for individual and diagnostic public-health use in RLS. Moreover, validation of wet-lab processes and data analysis pipelines is required for optimal detection and consistent interpretation of HIV resistance data for clinical utility. Ultimately, firm commitments and partnerships between the molecular diagnostics industry, local governments in RLS and global health agencies, are required to overcome HIVDR genotyping barriers that have often slowed down efforts by the UNAIDS to end the HIV epidemic [55].

## Figures and Tables

**Table 1:** Summary of popular NGS platforms.

Manufacturer	Platforms	Instrument Cost (US$)	Chemistry	Read Length (bp)	Maximum Output (Gb)	Error Rate (%)
Illumina	iSeq 100	19,900–950,000	SBS	150–300	0.3–6000	0.1
	MiniSeq					
	MiSeq					
	NextSeq					
	HiSeq					
	NovaSeq					
Thermo Fisher	Ion S5	60,000–149,000	Ion semiconductor	200–400	0.08–15	1
	Ion PGM					
	Ion Proton					
Pacific Biosciences	Sequel II	350,000–750,000	SMRT	60,000	0.5–10	13
	Sequel IIe					
	Sequel					
Oxford Nanopore Technologies	MinIon	1000–25,000	Nanopore	>100,000	10–960	12
	GridION					
	PromethION					

SBS, sequencing-by-synthesis; SMRT, Single Molecule Real-Time; US$, United States Dollars; bp, base pair; Gb, Gigabase. This table was adapted from the following references [12,22].

**Table 2:** In-house HIVDR genotyping assays for use in resource limited settings.

Year	Source	Country	Specimen Type	Vl Threshold	HIV-1 Gene	PMID
2006	Steegan K et al. [32]	Belgium	Plasma	≥500 cp/mL	PR, RT	16375980
2007	Chen JHK et al. [33]	China	Plasma	≥400 cp/mL	PR, RT	17449318
2008	Van Laethem K et al. [34]	Belgium	Plasma	NS	IN	18706932
2008	Pillay V et al. [35]	South Africa	Plasma	NS	PR, RT	18575198
2009	Hearps AC et al. [36]	Australia	Plasma	>50 cp/mL	IN	19917199
2009	Saravanan et al. [37]	India	Plasma	>1500 cp/mL	PR, RT	19490976
2010	Wallis CL et al. [38]	South Africa	Plasma	>1000 cp/mL	PR, RT	19917318
2010	Yang C et al. [39]	USA	DBS	<400 cp/mL	PR, RT	20660209
2011	Zhou Z et al. [30]	USA	Plasma and DBS	<400 cp/mL	PR, RT	22132237
2011	Fokam J et al. [40]	Cameroon	Plasma	>1000 cp/mL	PR, RT	21465085
2012	Chen JHK et al. [41]	Hong Kong	Plasma	≥400 cp/mL	PR, RT	22302906
2012	Parkin N et al. [42]	USA	DBS	≥1000 cp/mL	PR, RT	22544187
2013	To SWC et al. [8]	Hong Kong	Plasma	≥1000 cp/mL	IN	23886504
2013	Charturbhuj DN et al. [43]	India	Plasma	≥1000 cp/mL	PR, RT	23353551
2013	Aitken SC et al. [44]	Netherlands	Plasma and DBS	≥1000 cp/mL	PR, RT	23536405
2013	Inzaule S et al. [24]	Kenya	Plasma and DBS	≥1000 cp/mL	PR, RT	23224100
2014	Acharya A et al. [45]	India	Plasma	≥1000 cp/mL	PR, RT	25157501
2014	Manasa J et al. [29]	South Africa	Plasma	≥1000 cp/mL	PR, RT	24747156
2014	Chaturbhuj DN et al. [43]	India	Plasma	>1000 cp/mL	PR, RT	24533056
2015	Armenia D et al. [46]	Italy	Plasma	>50 cp/mL	IN	25712318
2017	Gupta S et al. [47]	Canada	Plasma	>100 cp/mL	PR, RT	28473986
2019	Seatla KK et al. [48]	Botswana	Plasma	>1000 cp/mL	IN	31751353
2020	Chrysostomou AC et al. [49]	Cyprus	Plasma	≥1000 cp/mL	PR, RT, IN	32061896

cp/mL, copies per microliter; DBS, dried blood spots; IN, integrase; NS, not stated; PR, Protease; RT, reverse transcriptase; SA, South Africa; VL, viral load.

**Table 3:** Strengths and limitations of Sanger sequencing and NGS in HIVDR genotyping methods.

	Strengths	Weaknesses
**Sanger sequencing**	Several validated methods for clinical use Low sequencing errors Relatively simple workflows and data analysis Relatively shorter turnaround times Common method in RLS Easily accessible software for interpretation (such as Stanford HIVdb)	High cost per test Cannot reliably detect LA-DRVs Not suitable for sequencing long genes/large genomes Not suitable for parallel testing High infrastructure costs Require standard molecular biology workflows
**Next generation sequencing**	Lower cost per test through pooling High sensitivity for LA-DRVs Suitable for sequencing long genes/large genomes Massive parallel sequencing	Only one validated method for clinical use (Sentosa HIV-1 genotyping kit) High sequencing errors Complex labor-intensive workflows and data analysis Longer turnaround time High infrastructure costs Requires specialized facilities No clear clinical significance of LA-DRVs Can produce unequal sequencing coverage Software for interpretation depend on data output files Requires personnel with high-level expertise Require standard molecular biology workflows

RLS, resource limited settings; DBS, dried blood spots; LA-DRV, low-abundance drug-resistant variants. This table was adapted from the following reference [14].

## Data Availability

Not applicable.
